# Novel Hybrid Feature Selection Using Binary Portia Spider Optimization Algorithm and Fast mRMR

**DOI:** 10.3390/bioengineering12030291

**Published:** 2025-03-14

**Authors:** Bibhuprasad Sahu, Amrutanshu Panigrahi, Abhilash Pati, Manmath Nath Das, Prince Jain, Ghanashyam Sahoo, Haipeng Liu

**Affiliations:** 1 Department of Information Technology, Vardhaman College of Engineering (Autonomous), Hyderabad 501218, Telangana, India; prasadnikhil176@gmail.com; 2 Department of Computer Science and Engineering, Siksha ‘O’ Anusandhan (Deemed to be University), Bhubaneswar 751030, Odisha, India; amrutansup89@gmail.com (A.P.); er.abhilash.pati@gmail.com (A.P.); 3 Department of Artificial Intelligence & Data Science, Vallurupalli Nageswara Rao Vignana Jyothi Institute of Engineering and Technology (VNRVJIET), Hyderabad 500090, Telengana, India; manmathnath.das@gmail.com; 4 Department of Mechatronics Engineering, Parul Institute of Technology, Parul University, Vadodara 391760, Gujarat, India; 5 Department of Computer Science and Engineering, GITA Autonomous College, Bhubaneswar 752054, Odisha, India; ghanarvind@gmail.com; 6 Centre for Intelligent Healthcare, Coventry University, Coventry CV1 5RW, UK

**Keywords:** cancer prediction, fast mRMR, binary Portia spider optimization (BPSOA), feature selection, weighted SVM

## Abstract

**Objective:** The cancer death rate has accelerated at an alarming rate, making accurate diagnosis at the primary stages crucial to enhance prognosis. This has deepened the issue of cancer mortality, which is already at an exponential scale. It has been observed that concentration on datasets drawn from supporting primary sources using machine learning algorithms brings the accuracy expected for cancer diagnosis. **Methods:** This research presents an innovative cancer classification technique that combines fast minimum redundancy-maximum relevance-based feature selection with Binary Portia Spider Optimization Algorithm to optimize features. The features selected, with the aid of fast mRMR and tested with a range of classifiers, Support Vector Machine, Weighted Support Vector Machine, Extreme Gradient Boosting, Adaptive Boosting, and Random Forest classifier, are tested for comprehensively proofed performance. **Results:** The classification efficiency of the advanced model is tested on six different cancer datasets that exhibit classification challenges. The empirical analysis confirms that the proposed methodology FmRMR-BPSOA is effective since it reached the highest accuracy of 99.79%. The result is of utmost significance as the proposed model emphasizes the need for alternative and highly efficient greater precision cancer diagnosis. The classification accuracy concludes that the model holds great promise for real-life medical implementations.

## 1. Introduction

Microarray technology is rapidly evolving, opening new avenues for molecular-level cancer research by generating massive gene expression databases. However, information knowledge differs from data. From these vast datasets, meaningful information must be extracted and biological data analyzed. Microarray gene expression datasets contain significant redundancy, are noisy, are high-dimensional, and have a small sample size. These qualities make feature selection more complex [1]. Many redundant genes may lead to a low categorization accuracy in general. Only a gene can be placed in a specific category using the conventional feature selection method based on the clustering method [2]. There is a biological condition that a gene can participate in several pathways, but this operation does not perfectly suit that requirement. In addition, using the threshold value to ascertain the quantity and dimensions of the modules cannot be considered appropriate. Methods such as independent and principal component analyses are based on the assumption that the original signal is statistically independent. Furthermore, it does not adhere to the internal interaction between transcription factors and biological genes. Because intelligent swarm optimization methods demonstrate greater performance, researchers have started employing them for feature selection [3]. However, because microarray gene expression datasets contain hundreds of genes, it is difficult to use an evolution algorithm to choose relevant genes directly related to the disease. As a result, combining two algorithms that complement one another for feature selection becomes a key area of research.

Authors [4] presented a hybrid two-stage model with the filter-based approach in the first stage to filter out unnecessary and unrelated features and then feed them into the wrapper method using the recent swarm-based algorithm, the salp swarm algorithm, SSA. In [5], a hybrid approach to feature selection is presented by combining an improved mRMR method with differential evolution. Here, the proposed approach modifies the quantization functions of mRMR to be compatible with the continuous features of microarray data that serve as an initial filter in the feature selection process. In addition, improved differential evolution is used to improve these properties. Alomari et al. [6] introduce a filtering technique called mRMR, which combines with a wrapping approach known as the Bat algorithm for gene selection in microarray data. The most significant genes were determined by using mRMR directly on the whole gene expression dataset set; conversely, BA was used to obtain the most relevant subset of genes from the reduced set created by MRMR to be used for cancer identification purposes [7]. This work combines an ensemble of filters that enhance the robustness and stability of mRMR. The filtering-based strategy generates discriminative genes. On the other hand, the wrapper-based strategy uses the findings from the filtering-based strategy to define the search space of gene selection. The wrapper approach encompasses the bat algorithm in gene selection while conjugating it with beta hill climbing, a potent local search method. It highlights the deep learning side when figuring out the search space and finding robust and stable discriminative genes. In [8], authors developed a feature selection approach called mRMR, which is integrated with an artificial bee colony (ABC) algorithm, which results in mRMR-ABC and also allows the identification of essential genes from the microarray profiles. An SVM algorithm is used to determine the classification accuracy of selected genes. Authors in [9] introduce a hybrid feature selection technique, mRMR-ICA, which will combine the minimum redundancy-maximum relevance (mRMR) with the imperialist competition algorithm (ICA) for cancer classification. The mRMR-ICA uses mRMR to remove redundant genes as a pre-feature selection step, thus providing ICA with reduced feature sets [10]. This paper presents a metaheuristic approach for optimizing the most relevant n genes from drug microarray data to improve the minimum redundancy-maximum relevance filter methodology. The study uses three metaheuristic algorithms: particle swarm optimization, cuckoo search, and artificial bee colony. Finally, k-nearest neighbors and Support Vector Machines are used as classifiers to reveal how effectively classification performance has been achieved [11]. The adopted approach will cover both filter and wrapper methodologies for selecting the biomarker genes. The set of worthy genes was obtained during the filtering step by applying MRMR (minimal redundancy and maximum relevance). The wrapper approach combines two principal metaheuristic approaches, BAT-HS and Support Vector Machine [12]. The new feature selection approach based on a combination of mRMR with an Aquila optimizer is presented. The mRMR methodology in the initialization stage improves the initial population qualities and improves the overall quality of the population. A random opposition-based learning strategy is superimposed over this base to enhance Aquila populations’ diversification with a faster algorithm convergence rate. A positional update equation of subsequent iterations of the Aquila optimizer is developed and added with inertia weight integration to avoid getting trapped into local optima and improve its detection capability for the global optimum [13]. The proposed approach is divided into two primary stages: the filter stage, with the mRMR attribute evaluation method, and the wrapper stage, with the strengths of the Northern Goshawk Algorithm (NGHA) and the SVM classifier for feature selection. Authors [14] discussed a new methodology called GradWise, which is proposed to improve the feature selection performance. GradWise introduces rank-based weighted hybrid filter-mRMR and the built-in feature selection technique to identify the most appropriate features from biomedical datasets. The author in [15] introduces a new feature selection model that ranks all features based on the mRMR feature selection technique. Features with higher ranking values are selected. In the following stage, the wrapper approach is used to select the best features. Such features are identified with the help of the Improved Equilibrium Optimization algorithm. The current study proposes a hybrid approach that integrates Salp Swarm Optimization (SSO) and Support Vector Machine (SVM) for breast tumor classification and gene selection. This methodology is applied in two steps: first, mRMR finds the informative and discriminative genes, and then SSO is applied to WSVM for classification. In SSO-assisted WSVM, redundant genes are eliminated, and important ones are weighted in the classification. SSO uses the weights of the selected genes in order to optimize kernel parameters [16]. The authors in [17] described and implemented a machine learning model for liver cancer diagnosis from expression data of genes. By unifying feature selection with a stacking ensemble learning model, the method addresses the issues of the high dimensionality and complexity of genomic data and improves diagnostic accuracy and interpretability. Similarly, in [18], authors designed a framework where the random parameters of an extreme learning machine (ELM) classifier are tuned using the self-adaptive multi-population-based elite strategy Jaya (SAMPEJ) algorithm. This results in a robust classifier, which is called SAMPEJ-ELM. In [19], authors propose and develop a two-stage ensemble deep learning methodology that utilizes metaheuristic approaches for detecting lung and colon cancers. This method consists of specialized deep CNN architectures for feature extraction and the electric eel foraging optimization algorithm to accurately diagnose a set of unique biomarkers through indicators for detection. A novel hybrid CSSMO metaheuristic learning-based approach has been developed, which utilizes a deep learning classifier for gene selection and correctly classifies cancer in cosmo-enabled spider monkey optimization and cuckoo search algorithm-selected genes, even for early-stage patients. The dimensionality of the gene expression data is further decreased by mRMR filtering, which improves the CSSMO outcome [20]. Deep learning (DL) techniques are also adopted by various researchers to optimize the features subset. In [21], a hybrid approach with deep learning technique called ES-DL. In the study, elephant search is used to select the optimal feature subset, followed by a stochastic gradient-based DL approach to reduce the feature subset. This study proposed a hybrid multifilter-ensemble model using the Grey Wolf Optimizer with two DL techniques like the Recurrent Neural Network (RNN) and Long Short-Term Memory (LSTM) classifiers for cancer prognosis [22]. In [23], to address the limitations that arise in machine learning algorithms, a hybrid approach with a combination of Enhanced Chimp Optimization (ECO) and different DL approaches are presented to deal with the cancer dataset.

### 1.1. Motivation and Objectives

The feature identification, of which the main characteristic is gene expression data, is the basis for improved diagnosis and therapeutic efficacy in identifying diseases. However, feature selection in gene expression data is prone to possible problems, such as poor removal of redundant features and suboptimal classification performance. This paper presents a novel feature selection method that aims to enhance the effectiveness of feature selection in high-dimensional gene expression datasets that exceed those adverse problems.

This work aims to present an enhanced feature selection method that utilizes fast mRMR and PSOA to enhance the discovery of critical features in gene expression data. Fast mRMR is a filter feature selection method grounded in information theory, adept at identifying the features most pertinent to the target function while minimizing redundancy across features. This study presents a strategy that effectively decreases the dimensionality of the features and improves the classification performance. The primary contributions of this paper are summarized as follows:

- Using fast mRMR in the PSOA initialization step in providing a better population-to-target optimization using feature significance, thereby forming a strong basis upon which subsequent optimization searches are built and in which the effectiveness of the algorithm in optimization is enhanced.
- Apply the PSOA optimization methodology in the process of selecting the most relevant features under a high-dimensional framework.
- Evaluate the performance of the proposed model using six distinct cancer microarray datasets.

### 1.2. Paper Structure

The rest of the paper is organized as follows. Section 2 describes some basic principles involved with fast mRMR and the Portia spider optimizer algorithm. Section 3 describes the implementation of the methodology presented. Section 4 carries out different studies to check the effectiveness of the presented strategy. This paper is concluded in Section 5.

## 2. Methods

### 2.1. Portia Spider Optimization Algorithm

PSOA is a recently developed swarm-enriched te chnique designed according to the hunting nature of Portia spiders. The design of the PSOA is carried out according to the following assumption while locating and attacking the preys [24].

- Portia spiders use the vibration released by the ensnared insects.
- A silk dragline is drawn by the Portia spiders to ensure the position of the prey.

Portia spiders use two different approaches for prey selection, which are presented below. In the first approach, to avoid the threat of neighbors, Portia spiders use the approach of chemical signals during prey selection. The position of the prey is identified according to the signal generated by the neighbor spider. The Portia spider then adjusts its movement to divert itself from the neighbors. After waiting a long time for the prey to focus, it gradually moves closer and attacks the prey. In the second approach, it identifies the prey using the natural web vibrations initiated by ambient breezes, else it has the potential to generate similar types of vibration independently. If the above-mentioned step fails to attract the prey, the Portia drags a silk dragline above the web and captures the prey. The adjustment of the position is performed dynamically according to the observed responses.

**Mathematical Design:** The framework conceptualizes and facilitates an improvement in a computational model that outlines the behaviors and strategies of Portia spiders. In this context, each Portia spider is an individual solution to a unique problem. Variables can be used to denote each spider or solution, possibly representing different parameters or characteristics of the solution.

(1) $$SP=\left[\begin{array}{cccc} sp_{1}^{1} & sp_{2}^{1} & \ldots & sp_{d}^{1}\\ sp_{1}^{2} & sp_{2}^{2} & \ldots & sp_{d}^{2}\\ \vdots & \vdots & \ddots & \vdots \\ sp_{1}^{N} & sp_{2}^{N} & \ldots & sp_{d}^{N} \end{array}\right]$$

To visualize the algorithm in detail, the expected solutions are presented in matrix format as mentioned in Equation (1). Each row of the representation is a Portia spider, or possibly a solution, while each column represents a solution variable or parameter. In Equation (1), *N* is defined as the total population of Portia spiders, and *d* denotes the variables or factors relevant to the optimization problem. This matrix comprises an all-inclusive view of the possible solutions and their corresponding characteristics. The positions of the Portia spiders that represent the solutions may oscillate throughout the performance of the algorithm, showing the optimization process or how the solutions come closer to the objective of the problem. The ranked fitness of Portia spiders is represented using Equation (2). The movement of Portia spiders depends on the position of the prey. The position of the prey is presented using Equation (3).

(2) $$SP=\left[\begin{array}{c} F(SP_{1})\\ F(SP_{2})\\ \vdots \\ F(SP_{N}) \end{array}\right]$$

(3) $$T=\left[\begin{array}{cccc} t_{1} & t_{2} & \ldots & t_{d} \end{array}\right]$$

For the optimal solution, the movement of Portia spiders, guided only by their prey’s position, becomes important. As if employing adaptive hunting strategies from Portia spiders, the PSOA navigates the solution space adaptively. This method allows the algorithm to employ some trial-and-error techniques and to adjust its strategies based on feedback, similar to how the Portia spiders refine their hunting tactics in response to the behavior of their prey. Unlike other metaheuristic algorithms, the PSOA algorithm needs a balance between exploration and exploitation. The details of the PSOA algorithm are presented using Algorithm 1.

- **Mechanism of stalking and striking (exploration phase):**
  This phase describes the position change mechanism of the Portia spider according to the chemical signal received from the neighbor spider. It changes myopathic gait and takes an instinctive jump to prevent the concealing of the prey. From this movement, the Portia spider confirms the position of the prey. The methodology behind this approach is mentioned using Equations (4) and (5).
  (4) $$SP_{ij}^{t+1}=\left\{\begin{array}{cc} t_{j} & \mathrm{if}\;\alpha_{1}<SPF\left(S_{i}\right),\\ SP_{ij}^{t} & \mathrm{if}\;\alpha_{1}\geq SPF\left(S_{i}\right), \end{array}\right.$$
  Subject to
  (5) $$SPF\left(S_{i}\right)=\frac{F(SP_{i})}{\sqrt{\sum_{1}^{N}F{\left(SP_{i}\right)}^{2}}}$$
  where $SP_{ij}^{t+1}$ presents the updated value of some state or variable $SP_{ij}$ at iteration t+1. $t_{j}$ depicts the best solution and *j*th the parameter of the targeted prey. The current value of $SP_{ij}$ in iteration *t* is symbolically presented as $SP_{ij}^{t}$. The random value of the variable $\alpha_{1}$ lies between 0 and 1. The standardized fitness score and the actual fitness score of the solution $SP_{i}$ are presented using $NF(SP_{i})$ and $F(SP_{i})$. The update rule can be interpreted as follows: If $\alpha_{1}$ is less than $SPF(S_{i})$, the value of $SP_{ij}$ is updated to a new candidate $t_{j}$. Otherwise, $SP_{ij}$ retains its previous value.
- **Mechanism of invading and imitating (exploitation phase):**
  This phase presents the natural techniques adopted by the Portia spider on the web of other spiders for easy hunting. In this stage, the Portia spider generates its own vibration and tries to sneak into the web of the prey. Rather than generating its own signal, it often takes advantage of the vibrations produced by the other insects like a gentle breeze. The intriguing skill of the Portia spider is presented using Equation (6).
  (6) $$sp_{ij}^{t+1}=r_{j}^{t}+\left|\alpha_{2}r_{j}^{t}-sp_{ij}^{t}\right|\times e^{\alpha_{3}}\times \cos\left(2\pi \alpha_{4}\right)$$
  Most Portia spiders adopt the above-mentioned trick to capture the prey. If it fails, a silk dragline is dragged just above the web so that it can reach the updated position and attack the prey with a single jump (new position change). The optimal solution or the new update position can be mathematically determined using Equation (7) from Equation (6).
  (7) $$sp_{ij}^{t+1}=r_{j}^{t}+\alpha_{2}\times \left(r_{j}^{t}-sp_{ij}^{t}\right)$$

The final exploitation phase can be derived using Equation (8) to clearly understand the mechanism behind this study.

(8) $$sp_{ij}^{t+1}=\left\{\begin{array}{cc} r_{j}^{t}+\left|\alpha_{2}r_{j}^{t}-sp_{ij}^{t}\right|\times e^{\alpha_{3}}\times \cos\left(2\pi \alpha_{4}\right) & \alpha_{5}<0.5\\ r_{j}^{t}+\alpha_{2}\times \left(r_{j}^{t}-sp_{ij}^{t}\right) & \alpha_{5}\geq 0.5 \end{array}\right.$$

Subject to

(9) $$\alpha_{3}=1-{\left(\frac{I_{cur}}{I_{max}}\right)}^{2}$$

Here, $\alpha_{1}$, $\alpha_{2}$, and $\alpha_{3}$ are three random variables and the values lies within the range 0,1. To balance the gap between both phases, the value of $\alpha_{3}$ is calculated using Equation (9), where $I_{cur}$ and $I_{max}$ denote the current iteration and maximum iteration, respectively.

The following observations of PSOA encourage us to design a new binary PSOA present in this study to propose a new hybrid model called mRMR-BPSOA. The choice of BPSOA is based on its unique problem-solving strategies, inspired by the predatory behavior of Portia spiders, which provide significant advantages over conventional metaheuristics. PSOA addresses issues with PSO, GA, and ACO-style heuristics suffering from premature convergence by incorporating tactical hunting and memory-based tracking, maintaining a balance between exploration and exploitation. By employing Portia spider-inspired probabilistic movement patterns, BPSOA mitigates stagnation in suboptimal regions. Unlike PSO’s reliance on velocity updates and GA’s crossover and mutation dependency, PSOA enhances global optimality detection with intelligent searching strategies. Microarray gene expression data sets present complex problems for optimization algorithms, especially in feature selection with multiple dimensions. PSOA sustains robustness in multidimensional feature spaces due to its dynamic prey tracking and adaptive step-size mechanisms. Ant Colony Optimization, Whale Optimization Algorithms, and other bio-inspired algorithms face high computational costs due to the extensive function evaluations they require. Through selective strategic engagement and memory retention, PSOA limits unnecessary evaluations, enhancing execution speed without compromising solution quality. Algorithm 1 shows the working of PSOA.
**Algorithm 1** Portia spider algorithm**Require:** Population size (*N*); number of iterations ($I_{\max}$)
1:**Begin**2:Generate random Portia spiders3:**while** $I_{\mathrm{cur}}<I_{\max}$ **do**4:    Calculate and sort the fitness value;5:    Determine prey position;6:    *exploration phase*;7:    Update value of $\alpha_{1}$;8:    Calculate standardized fitness score;9:    Update Portia spider position using Equation (4);10:    *exploitation phase*;11:    Update values of $\alpha_{2}$, $\alpha_{3}$, $\alpha_{4}$, and $\alpha_{5}$;12:    Update Portia spider position using Equation (8);13:**end while**14:Update Portia spider set;15:Determine the best solution;16:$I_{\mathrm{cur}}=I_{\mathrm{cur}}+1$;
**Ensure:** Optimal solution and its fitness score.


### 2.2. Fast mRMR

Among filter-based feature selection methods, one widely applied approach is the mRMR algorithm first introduced by Peng et al. [25]. It calculates the relevance of the features toward the class label using mutual information, as well as the probability that they are redundant with each other. The technique was first invented for the evaluation of microarray gene expression data, which performed excellently. It is, however, currently applied in many fields, such as medicine and anomaly detection in networks and telecommunications. Although the above explanation emphasizes the application to discrete data distributions, the method can also be applied to continuous data, as formulated in the initial description of this method.

mRMR is a sophisticated algorithm designed with the explicit objective of ranking all possible features based on their relevance and importance in relation to that feature [26]. This ranking may be achieved through the evaluation of how much each individual feature may be related to and relevant to the target variable of interest, along with the integration of a mechanism by which the features are penalized for redundancy or one feature overlapping with another. In this paper, a concept called mutual information (I) is used, in which the stated primary objective is to maximize the level of dependency and connection associated with the set of features X with the corresponding class c. This objective will be met by mutual information. Using mutual information, by measuring the marginal probabilities that are defined as p(a) and p(b), and the joint probability, which is defined as p(a,b), it is a measurable quantity describing the strength of dependence or the relationship between two discrete random variables, also known as features, expressed in Equation (10) through the marginal probabilities mentioned p(a) and p(b).

(10) $$I\left(A;B\right)=\sum_{b\in B}\sum_{a\in A}p\left(a,b\right)\log\left(\frac{p(a,b)}{p(a)p(b)}\right)$$

This could then be approximated based on available data samples in case marginal probabilities are not defined. In fact, Equation (2) supports the same principle, where *n* represents the number of samples which take the value $n_{a}$ for the feature in question and *n* is the total number of samples. For example, to show this, consider the case as *p*(*a*,*b*).

(11) $$p\left(a\right)\approx \frac{n_{a}}{n}$$

Similarly, using Equation (12), we can estimate the joint probability where the values of the desired characteristics are indicated by *a* and *b*, and the number of samples that include *a* and *b* in those features is denoted by $n_{a,b}$.

(12) $$p\left(a\right)\approx \frac{n_{a,b}}{n}$$

In view of the difficulties involved in applying the maximum dependency criterion in high-dimensional environments, maximal relevance (max-relevance), as defined in Equation (13), was used in replacement in the original proposal. Here, a set of distinguishing features is symbolized by *X*.

(13) $$\max D\left(X,c\right),D=\frac{1}{\left|X\right|}\sum_{x_{i}\in X}I\left(x_{i};c\right)$$

The chosen attributes may be highly correlated with each other; therefore, they are included as a penalty to allow for mutual exclusive selection. The minimum redundancy criterion is defined as Equation (14). In that definition, *X* represents a set of features.

(14) $$\min R\left(X\right),R=\frac{1}{{\left|X\right|}^{2}}\sum_{x_{i},x_{j}\in X}I\left(x_{i};x_{j}\right)$$

The computational efficiency of the fast mRMR is O(N) and it uses the matrix-based model whereas the traditional mRMR uses the pair-wise MI method and performs with computational efficiency of $O(N^{2})$. In many studies, traditional mRMR is not adequate while dealing with high-dimensional datasets. So, the updating scheme and matrix transformation architecture of fast mRMR can deal the high-dimensional datasets with low overhead. Fast mRMR applies advanced optimization techniques, whereas classical mRMR uses an iterative approach to deal with redundancy which affects the performance of the classifier. Fast mRMR effectively handles high-dimensional datasets by prioritizing relevant features without excessive computational burden in comparison with traditional mRMR.

High computational cost, dependence on data quality, inability to handle complex feature interactions, scalability issues, and a lack of adaptation to continuous data are a few limitations raised with the basic mRMR. So, in this study, we have used fast mRMR. The algorithm then calculates the relevance value for each input feature and stores it inside a vector named relevance vector. The algorithm then decides which features are most relevant to the problem at hand and declares the feature with the highest relevance to be the last selected feature. The remaining features then start getting selected one after the other under the supervision of fast mRMR criterion. This process is to be repeated until the number of features required is obtained. Then, at each iteration of this iterative procedure, the algorithm computes how much mutual information is between the last selected feature and the features that are yet to be selected. Algorithm 2 shows the working of Fast mRMR feature selection. Most importantly, the most valuable feature, according to mRMR, is included in the final selection and is denoted as the feature selected last. The accumulated redundancy vector is one of the improvements included in this methodological framework. It is expensive, and an accumulation of redundancy occurs at each step and only information about the mutual information between the set of features that were not selected and the most recently selected feature is computed. This is because it computes mutual information between every possible pair in detail [27].
**Algorithm 2** Fast mRMR feature selection**Require:**1:Dataset with *n* features: $F=\{f_{1},f_{2},\ldots ,f_{n}\}$2:Target variable *T* (class label)3:Number of features to select: *k***Ensure:** Selected subset of *k* features: S⊆F
4:**Initialize:** S={}                   ▹ Empty feature subset5:**Compute Relevance:**6:**for** each feature $f_{i}\in F$ **do**7:    Calculate mutual information $I(f_{i};T)$8:**end for**9:**Iterative Feature Selection:**10:**while** |S|<k **do**11:    **Select Feature with Maximum Relevance:**12:    Select $f_{i}\in F\setminus S$ with highest $I(f_{i};T)$13:    **Compute Redundancy:**14:    **for** each feature $f_{j}\in F\setminus S$ **do**15:        Calculate $R\left(f_{j};S\right)=\frac{1}{\left|S\right|}\sum_{f_{s}\in S}I\left(f_{j};f_{s}\right)$16:    **end for**17:    **Evaluate mRMR Criterion:**18:    **for** each feature $f_{j}\in F\setminus S$ **do**19:        Calculate $\mathrm{mRMR}\left(f_{j}\right)=I\left(f_{j};T\right)-R\left(f_{j};S\right)$20:    **end for**21:    **Add Feature to Subset:**22:    Select feature with maximum mRMR score23:    Add selected feature to *S*24:**end while**25:**return** Selected feature subset *S*


### 2.3. Binary Portia Spider Optimizer Algorithm

Such a traditional PSOA can be used only in problems of continuous value optimization and cannot be applied to discrete values. The feature selection problem can be imagined as a binary vector that consists of only 0s and 1s, where 0 marks the exclusion of the feature and 1 shows its inclusion. This paper introduces a sigmoid transfer function to transform the values from the continuous search space into a discrete one, represented by Equation (15):

(15) $$S\left(X\right)=\frac{1}{1+e^{-10(X-0.5)}}$$

Here, *X* and S(X) present the original and probability binary value of *X* after conversion. So, the new position of the Portia spider can be expressed using Equation (16).

(16) $$SP_{ij}\left(t\right)=\left\{\begin{array}{cc} 1, & \mathrm{if}\;S(SP_{ij}\left(t\right))>\mathrm{rand}\\ 0, & \mathrm{otherwise} \end{array}\right.$$

BPSOA exploits the efficient Portia spiders’ hunting behavior for local optimal escape and broadened search space. The algorithm captures the essence of adaptive predation by allowing candidate solutions to change their movements based on feedback from the environment. By enabling the BPSOA to remember previously found solutions, it becomes possible to improve them over successive cycles, thus avoiding premature convergence. This approach improves the balance between exploitation and exploration by focusing on good regions while preventing stagnation. Moreover, movement based on the Portia spider’s erratic style of hunting ensures that diverse and less constrained search behavior is realized, which mitigates the chances of being stuck in local optima. Deceptive fitness landscapes are helped by the use of stochastic perturbations and variable step sizes. Collectively, these actions increase the perceived stability of convergence and the likelihood of finding global optima in high-dimensional space.

### 2.4. Weighted Support Vector Machine

SVM is a strong supervised machine learning algorithm that is used in classification and regression problems. It works like the creation of an optimal hyperplane, maximized to create a margin between two classes in a given data set. Margin refers to the distance between the closest points of each class to the hyperplane, which are called support vectors. At a higher level of abstraction, the SVM algorithm is designed to create a boundary between classes that maximizes separation with an eye toward maximizing generalization to new unobserved instances [28].

SVM can be used to solve non-linear and linear classification problems. For separable and linear data, it develops a linear hyperplane. It uses kernel functions, including the RBF, polynomial, and sigmoid kernels for non-linear data. These kernels map the data in a high-dimensional space so that it is possible to implement the linear hyperplane. The method is called the kernel trick, where SVM can efficiently address complex classifications without applying the direct execution of the transformation.

The regularization parameter and the gamma parameter are the significant parameters of SVM, which regulate the trade-off between maximizing the margin and minimizing the error during classification. However, one of the significant limitations of conventional SVM is that it assumes that all the genes have a homogenous contribution to increase the accuracy of classification. The present paper proposes a weighted Support Vector Machine, which integrates PSOA.

The weighted Support Vector Machine (weighted SVM) is an extension of the basic SVM algorithm. It has been specially customized to handle imbalanced datasets. In datasets, if one class is significantly larger compared to the others, then the standard SVM is highly biased to favor the larger class. The classifier performs poorly for classification in the case of smaller classes. Weighted SVM addresses this issue by assigning different weights to the classes in question and penalizing errors more heavily for the minority class compared to the majority class. In weighted SVM, different penalty weights ($C^{+}$ and $C^{-}$) are assigned to the majority and minority classes, respectively:

(17) $$\min_{w,b,\xi }\frac{1}{2}{∥w∥}^{2}+\sum_{i=1}^{n}C_{i}\xi_{i}$$

where $C_{i}$ is a class-specific weight defined as:

(18) $$C_{i}=\left\{\begin{array}{cc} C^{+}, & \mathrm{if}\;y_{i}=+1\;(\mathrm{minority}\mathrm{class}),\\ C^{-}, & \mathrm{if}\;y_{i}=-1\;(\mathrm{majority}\mathrm{class}). \end{array}\right.$$

The constraints remain the same:

(19) $$y_{i}\left(w^{⊤}x_{i}+b\right)\geq 1-\xi_{i},\;\xi_{i}\geq 0,\;i=1,2,\ldots ,n.$$

The dual form of the weighted SVM becomes:

(20) $$\max_{\alpha }\sum_{i=1}^{n}\alpha_{i}-\frac{1}{2}\sum_{i=1}^{n}\sum_{j=1}^{n}\alpha_{i}\alpha_{j}y_{i}y_{j}K\left(x_{i},x_{j}\right)$$

subject to:

(21) $$0\leq \alpha_{i}\leq C_{i},\;\sum_{i=1}^{n}\alpha_{i}y_{i}=0,$$

where $K(x_{i},x_{j})$ is the kernel function used to handle non-linear decision boundaries.

The selection of WSVM in our study is motivated by its superior handling of imbalanced data, the evaluation of the importance of characteristics and the robustness of classification.

## 3. FmRMR-PSOA: The Proposed Model

**Figure 1:**
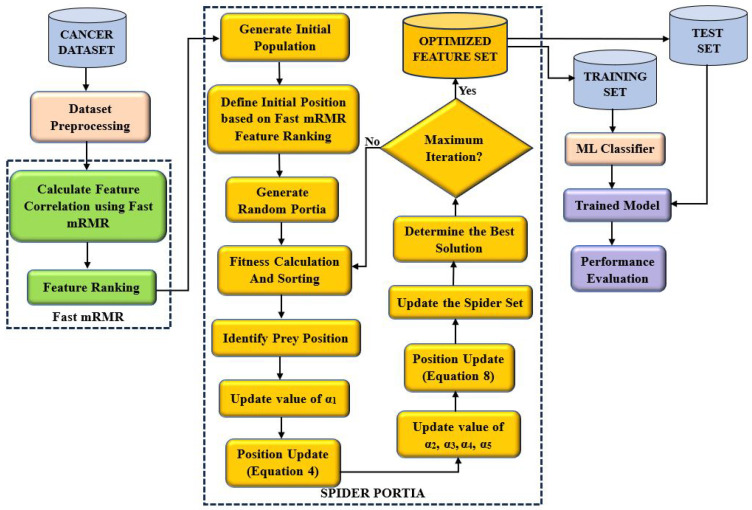
Workflow model of FmRMR-BPSOA.

This section proposes a new feature selection method known as FmRMR-PSOA. In the first phase, the fast mRMR filter algorithm is applied to select the appropriate features with an enhancement in population diversity through the initialization of two distinct populations. After that, three different methods are employed to improve the search efficiency of the binary PSOA technique, and at the final stage, an optimal feature subset is achieved with high accuracy and a smaller number of features. Finally, the appropriate evaluation metric was obtained in the test set by performing a 5-fold cross-validation of the best subset of characteristics to illustrate the performance of this subset of characteristics. Figure 1 illustrates the flow chart related to FmRMR-PSOA.

**Figure 2:**
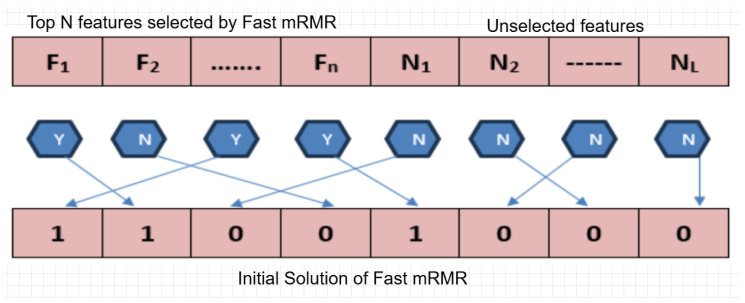
Initial population of fast mRMR.

The initialization method of population vectors in BPSOA is very important. In the conventional BPSOA approach, it generates members of the initial population by picking random specific characteristics. Even though it gives a broad spectrum of possibilities and converges towards feasible solutions to a great extent, the initial search phase of BPSOA takes tremendous computational time as classifiers have to be repeatedly evaluated for the best solutions. This paper utilizes fast mRMR as an extraction of features, which enhances the velocity of convergence of BPSOA. We propose a dual method for the initial population by making use of the result of fast MRMR. The initial members are created using the set of relevant features, as obtained from fast MRMR. The initial population of fast mRMR is presented in Figure 2.

A novel variant of the binary BPSOA method tailored specifically for the feature selection task in microarray data analysis. PSOA is a global adaptive optimization algorithm that is commonly used because it is simple to implement, converges quickly, and is quite robust. It has been successfully applied to various fields, including data mining, pattern recognition, and artificial neural networks. We enhance the PSOA algorithm by introducing a new binary quantization approach and scaling factor. This update will further enhance the exploratory capability of the algorithm in the exploration phase and enhance population diversity. In addition to this, we also guarantee that the exploitation capability of the algorithm for the next phase will exploit local optima. We present an adaptive crossover operator designed to improve the speed of convergence in the initial phase while maintaining the algorithm’s ability to exploit solutions in later stages. This paper introduces a new advanced binary PSOA technique that effectively balances exploration and exploitation to solve the feature-selection problem of microarray data. This improvement provides more efficient and accurate feature relevance detection for the assessment of microarrays, which improves the general efficiency and effectiveness of the feature selection procedure. The mechanism behind the execution of BPSOA is presented in Section 2. BPSOA exploits the efficient Portia spiders’ hunting behavior for local optimal escape and broadened search space. The algorithm captures the essence of adaptive predation by allowing candidate solutions to change their movements based on feedback from the environment. By enabling the BPSOA to remember previously found solutions, it becomes possible to improve them over successive cycles, thus avoiding premature convergence. This approach improves the balance between exploitation and exploration by focusing on good regions while preventing stagnation. Moreover, movement based on the Portia spider’s erratic style of hunting ensures that diverse and less constrained search behavior is realized, which mitigates the chances of being stuck in local optima. Deceptive fitness landscapes are helped by the use of stochastic perturbations and variable step sizes. Collectively, these actions increase the perceived stability of convergence and the likelihood of finding global optima in high-dimensional space.

The feature selection aims at achieving a subset of features from dataset X, which would result in maximum classification accuracy using limited feature information. However, we could not get most of these used datasets balanced; hence, the accuracy had to be replaced with MCC for this study. Of course, each of the above factors has different contributions with respect to the effectiveness of the KNN classifier in a given classification. We combine all of this into a single weighted metric and apply the same fitness function. The fitness function used in this study is presented in Equation (22).

(22) $$F=\alpha \times \mathrm{MCC}\left(\mathrm{KNN}\right)+\left(1-\alpha \right)\times \left(1-\frac{N_{f}}{N}\right)$$

where *N* and $N_{f}$ depict the total number of features and features selected. The constant α represents the weight of the associated objective, and the value is between [0,1].

### 3.1. Implementation Steps of fastmRMR-BPSOA

In the context of the proposed model FmRMR, the following is the process of Feature selection using fast mRMR and PSOA. Fast mRMR is used for ranking the features, and PSOA is used to select the optimal set of features from the dataset.

**Input:**

- Dataset *D* with features $F=\{f_{1},f_{2},\ldots ,f_{n}\}$ and labels *Y*. [Training data (80%)-Test data (20%)].
- Number of top features *M* to consider after fast mRMR.
- PSOA parameters: population size *N*, maximum iterations *T*, and attraction–repulsion parameters.

**Output:**

- Optimal subset of features $F_{\mathrm{opt}}$.

**Steps:**

1. **Apply fast mRMR**
  (a) Compute the mutual information (MI) for all features with respect to the target variable *Y*.
  (b) Apply fast mRMR to rank the features based on their relevance to *Y* and minimal redundancy among features.
  (c) Select the top *M* features: $F_{\mathrm{top}}=\left\{f_{1},f_{2},\ldots ,f_{M}\right\}$.
2. **Initialize PSOA**
  (a) **Representation:** Each spider is a binary vector $S_{i}=\left\{b_{1},b_{2},\ldots ,b_{M}\right\}$, where $b_{j}=1$ indicates inclusion of feature $f_{j}$, and $b_{j}=0$ indicates exclusion.
  (b) Randomly initialize a population of *N* spiders $\left\{S_{1},S_{2},\ldots ,S_{N}\right\}$. Each vector has a size of *M*, corresponding to the features in $F_{\mathrm{top}}$.
3. **Define Fitness Function**
  (a) For each spider $S_{i}$:
    i Extract the feature subset $F_{\mathrm{sub}}$ corresponding to $S_{i}$.
    ii Train a machine learning model ML using $F_{\mathrm{sub}}$ on training data.
    iii Evaluate ML using a validation metric (e.g., accuracy, F1 score).
    iv Fitness score: $\mathrm{Fitness}\left(S_{i}\right)=\mathrm{Evaluation}\;\mathrm{Metric}\left(ML\right)$.
4. **Run PSOA**
  (a) **Iteration:** For t=1 to *T*:
    i For each spider $S_{i}$:
      A. Update its position (binary vector $S_{i}$) based on PSOA attraction–repulsion rules and fitness scores of other spiders.
      B. Apply boundary constraints to ensure binary values {0,1}.
    ii Evaluate fitness for updated positions of all spiders.
  (b) **Stopping criteria:** Stop if the algorithm converges (no significant improvement in fitness) or reaches *T*.
5. **Return the Optimal Subset**
  (a) Identify the spider $S_{\mathrm{best}}$ with the highest fitness.
  (b) Return $F_{\mathrm{opt}}$, the feature subset represented by $S_{\mathrm{best}}$.

### 3.2. Outline of Classifiers

The use of several classifiers enables the article to achieve the research goals. This is especially true for basic algorithms like SVM and DT, and for ensemble learning-based methods which include two boosting algorithms, XGBoost and AdaBoost, as well as a bagging method, RF [29,30]. These algorithms are briefly discussed in this section.

#### 3.2.1. Support Vector Machine (SVM)

The objective of the Support Vector Machine (SVM) is to construct a hyperplane that maximizes the margin between classes in the dataset. This hyperplane is given by Equation (23).

(23) $$H:\omega^{T}\times x+d=0$$

Select the subset of features with the highest fitness score [24].

The letter w is a normal vector for the concerned hyperplane and d represents the distance of the hyperplane from a point (*x_i_*, *y_i_*). The hyperplane is represented by the superplane. The dual problem can be obtained through the Lagrange multiplier application as given in Equation (24).

(24) $$L=\frac{1}{2}{∥\omega ∥}^{2}-\sum_{i=1}^{n}\kappa_{i}y_{i}\left(\omega \cdot x_{i}+d\right)+\sum_{i=1}^{n}\kappa_{i}$$

Feature selection is a minimal problem. So, the value of $\frac{\delta L}{\delta x}$ is equivalent to 0. So, the value of ω can be expressed in Equation (25).

(25) $$\omega =\sum_{i=1}^{n}\kappa_{i}\cdot y_{i}$$

#### 3.2.2. Decision Tree

The decision tree is a widely used machine learning technique that is trained on a dataset for classification and regression analysis. This model classifies samples based on answers to many questions. A tree is an excellent example of the classification process. The first level of the tree includes all samples. The most difficult part of creating a decision tree is the choice of optimal partition characteristics, which can be estimated with the help of entropy, gain ratio, or gini index.

#### 3.2.3. XGBoost

Extreme GB (Gradient Boosting) has advanced benefits proprietary to addressing a knowledge base problem. It harnesses the fixed boosting algorithms which are very accurate and emphasize scrimped data control. This also makes it applicable to large feature datasets. This technique also happens to be one of the best implementations of a gradient-boosted decision tree (gbDT), which has become a centric topic because of its rapid speed and ease of model execution. The trees estimate weight in order to get close to the goal variable and take a number of actions at every leaf node. A new method was presented that employs boosting processes to aid in combining the predictive power of weak or base component classifiers into one strong classifier. One variety of component classifiers is a decision tree. This model is popularly used in many areas such as regression, ranking and classification because of its unmatched flexibility and agility in computing. XGBoost, the most popular ensemble algorithm for gradient-boosted learning provides a good estimate for the target variable. It originates from innovative works based on information from newly constructed trees. In every iteration, the incorrectly classified points are evaluated by examining the most recent tree. In addition, these points are assigned weights. The next cycle focuses on creating a new tree which helps in correctly classifying the points mentioned earlier. In this manner, a new tree is acquired after each cycle. The final model uses an ensemble of all trees generated in previous cycles to minimize the misclassification error. With this method, the entire process is additive and self-reinforcing. As a start, the model was built using the default parameters.

#### 3.2.4. AdaBoost

AdaBoost was originally conceived by Freund and Schapire with the aim of enhancing the classification accuracy of a base algorithm. They accomplish this by employing a methodology in which a set of weak classification functions attempts to build a more powerful classifier. Their boost strategy is not only effective for achieving high classification speed but also serves as an alternative for feature selection.

In particular, Adaboost is considered the most widely used boosting strategy because it captures the intuition of boosting perfectly. Adaboost has wide applicability and is one of the most effective boosted approaches with sequential learning. To select the best subset from the base classifier pool, it picks the most important weak classifiers and combines them into a strong classifier.

In the realm of boosting, AdaBoost, which was designed by Freund and Schapire in 1997, is considered to be the most robust machine learning technique. In addition, it can be regarded as a primary stepping stone having great utility for classification tasks. This method did not only help solve the problem of weak learners but also single-handedly solved the problem of overfitting and served as a fast yet effective classifier. An added advantage of this system is that the features are selected based on the previously selected weak classifiers. The classifiers that are initially selected as stumps, or classifiers containing a single decision split, are indicative of weak counters.

The idea of having a single source (root) node attached to a pair of nodes is pictured as a decision stump which is built for every feature of given data. Essentially, every AdaBoost implemented is considered as a unique trained repeating algorithm that performs like so: AdaBoost is provided with a unique set of training instances (samples), which, over the course, is achieved by changing the partition of weight over training instances (samples). In the end, alongside the weighted mean of a multitude of soft classifiers, a single robust classifier is said to be obtained. AdaBoost performs well with ensemble classifiers that are robust, or at least, it is capable of building those types of classifiers. It does not ensure a reduction in mean error, which is rather problematic. Because of using the ensemble of classifiers, the mean error increases. This is the reason why it succeeds. In order not to “weaken” it too much, and for the refinement of the last ensemble, the set of various weak classifiers is used. Basically, the rest of AdaBoost brings an example of an autonomous algorithm that boasts effectiveness yet falls short of perfect precision.

#### 3.2.5. Random Forest

The ensemble technique Random Forest (RF) is made up of many decision trees that are trained and their outputs are combined for better prediction accuracy and less overfitting. Each tree in the forest is trained on different subsets of the data using the bagging technique. This helps the trees gain diversity among them. Each tree outputs a classification and they are aggregated through majority voting. For classification and regression tasks, Random Forest is very efficient due to its accuracy, capability of handling overfitting considerably better than individual decision trees, and tackling high-dimensional data.

### 3.3. Summary of Biomedical Datasets

This paper utilizes six publicly available biomedical datasets to evaluate the performance of the fastmRMR-BPSOA algorithm relative to the performance achieved by other methods. The datasets that were used for ALL-AML (A1), colon tumor (A2), central nervous system (A3), and ovarian cancer (A4) are available in a single zip file for download from the website http://csse.szu.edu.cn/staff/zhuzx/Datasets.html accessed on 14 December 2024. The other two datasets, GSE4115 (A5) and GSE10245 (A6), can be obtained from GEO database at https://www.ncbi.nlm.nih.gov/geo accessed on 14 December 2024. The NCBI has maintained GEO which is a central biological database which contains large volumes of gene expression data that are contributed to by different researchers around the world.

The datasets used in the experiments differ in size: their instance numbers range from 60 to 235, and all are of high dimensionality; the features vary between 2000 and 54,676. These datasets provide valuable information on gene expression, protein profiling, and genomic sequence, which can be used for classification and disease diagnosis purposes. Table 1 gives a summary of the details of these biomedical datasets.

## 4. Experimental Analysis

This part describes a set of experiments carried out to test the performance and effectiveness of the proposed method. First, the raw datasets were examined in order to establish a performance measure for classification in the absence of any feature selection, which served as a basic measure in such analyzes. Then, the BPSOA algorithm was tested using these raw datasets in order to evaluate its capacity in feature selection. Finally, the outcomes achieved with BPSOA were compared to the non-BPSOA outcome from the primary datasets.

The new hybrid system was applied to competitions to check how they would perform against the other examined hybrid systems in this work. It started using the fast mRMR approach, which was a first-pass filter designed to increase the performance of a particular learning algorithm by eliminating non-informative features from the datasets. The resultant reduced datasets were then input into the wrapper phase, where BPSOA was used to initialize the search space. To evaluate the approach, five classifiers were applied, basically including simple models, SVM, weighted SVM and DT, as well as more sophisticated ensemble strategies like XGBoost AdaBoost (boosting) and Random Forests (bagging). Classifier performance was foremost evaluated in terms of accuracy, which is the fraction of the correctly classified test instances, which is appropriate for the research question and is the use of the fitness function.

The splitting of the datasets is carried out such that 80% was used for training and 20% for testing. The hybrid framework is developed using a system with a Windows 10 OS, equipped with an Intel i7 processor with a 4.6 GHz clock speed, 16 GB of RAM, 1 TB of SSD, and 1 TB of HDD. The analysis of the proposed model is evaluated in two different phases. In the first phase, the model is made without using the BPSOA algorithm and in the second phase of the evalution the model is evaluated using the BPSOA algorithm. To evaluate the performance of the developed models different parameters Accuracy $\left(A_{cc}\right)$, Misclassification Ratio (MCR), Precision $\left(P_{re}\right)$, Recall $\left(R_{cl}\right)$, Specificity $\left(S_{pe}\right)$, F1 Score $\left(F_{1Sc}\right)$, False Negative Ratio $\left(Fa_{Neg}\right)$, False Positive Ratio $\left(Fa_{Po}\right)$, and Mathews Correlation Coefficient $\left(M_{CC}\right)$ are considered. Equations (26)–(33) show the calculation of the above mentioned parameters with $t_{Po}$, $f_{Po}$ as true and false positive, respectively, $t_{Ne}$, $f_{Ne}$ as true and false negative, respectively.

(26) $$A_{cc}=\frac{t_{Po}+t_{Ne}}{t_{Po}+f_{Po}+t_{Ne}+f_{Ne}}$$

(27) $$P_{re}=\frac{t_{Po}}{t_{Po}+f_{Po}}$$

(28) $$R_{cl}=\frac{t_{Po}}{t_{Po}+f_{Ne}}$$

(29) $$S_{pe}=\frac{t_{Ne}}{t_{Po}+f_{Ne}}$$

(30) $$F_{1Sc}=\frac{2\times \frac{t_{Po}}{t_{Po}+f_{Po}}\times \frac{t_{Po}}{t_{Po}+f_{Ne}}}{\frac{t_{Po}}{t_{Po}+f_{Po}}+\frac{t_{Po}}{t_{Po}+f_{Ne}}}$$

(31) $$Fa_{Neg}=\frac{f_{Ne}}{t_{Po}+f_{Ne}}$$

(32) $$Fa_{Po}=\frac{f_{Po}}{t_{Po}+f_{Ne}}$$

(33) $$M_{CC}=\frac{\left(t_{Po}\cdot t_{Ne}\right)-\left(f_{Po}\cdot f_{Ne}\right)}{\sqrt{\left(t_{Po}+f_{Po}\right)\left(t_{Po}+f_{Ne}\right)\left(t_{Ne}+f_{Po}\right)\left(t_{Ne}+f_{Ne}\right)}}$$

### 4.1. Phase-I Evaluation

**Figure 3:**
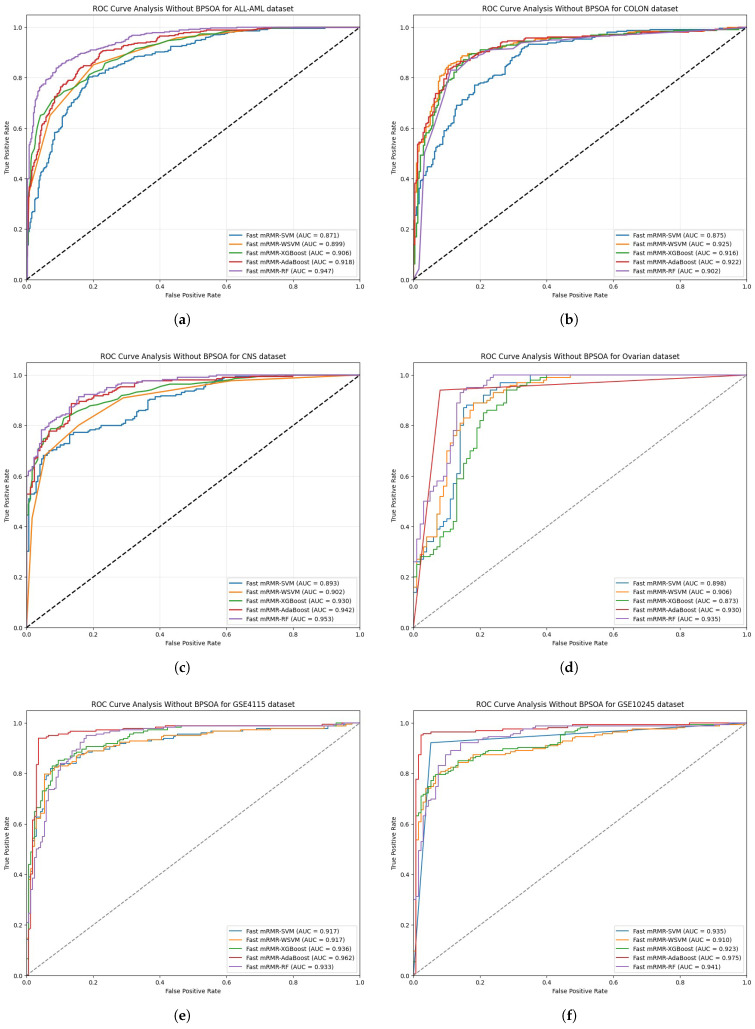
ROC analysis for (**a**) ALL-AML (**b**) colon (**c**) CNS (**d**) ovarian (**e**) GSE4115 (**f**) GSE10245 cancer datasets without using BPSOA as the feature selection algorithm.

- Out of all the methods, fast mRMR-RF outperformed the others at 93.50% in the ALL-MLL dataset, achieving the highest accuracy. fast mRMR-RF surpassed the next best model, fast mRMR-AdaBoost, by 0.61%. It achieved an F1-Score of 94.78 and an MCC of 86.60. Compared to the worst algorithm of fast mRMR-SVM, the Random Forest algorithm was found to outperform by 4.61%. Additionally, it achieved an FNR of 1.67%, indicating an impressive ability to predict true positives with an FPR of 13.75%.
- Fast mRMR-AdaBoost had the best results in the colon cancer dataset with an accuracy of 93.94% and an F1-Score of 95.24. This was an improvement of 0.51% over the previous best algorithm fast mRMR-WSVM. Compared to fast mRMR-SVM, which had an accuracy of 88.38%, AdaBoost demonstrates an improvement of 5.56%. Moreover, with an MCR of 6.06%, AdaBoost was also able to balance sensitivity at 97.56% with specificity at 88%, pointing toward an impressive reduction in misclassifications.
- In the case of the central nervous system dataset, the implemented model fast mRMR-RF has the best performance with 93.43% accuracy while qualifying F1-Score at 94.65%. It surpasses the second-best model, which is fast mRMR-AdaBoost, by a margin of 1.01% in accuracy and improves the fast mRMR-SVM method by 6.06%. Its MCC is 86.29%, combined with the lowest FPR of 11.39% makes the model Random Forest demonstrate strong classification results with low false alarms. The model creates these results while having an MCR that is low at 6.57%.
- Regarding the ovarian cancer dataset, the achieved results are different as the model fast mRMR-AdaBoost exhibits the best performance with an achieved overall accuracy of 92.93% while meanwhile having an F1-Score of 94.89. This model shows improvement of 1.01 in comparison with the second-best model fast mRMR-XGBoost while increasing 3.03% over fast mRMR-SVM. With 87.10% specificity combined with an FNR of 4.41%, the model AdaBoost demonstrates performance that is stable and predictive for this dataset.
- As indicated in GSE4115 dataset, the fast mRMR-AdaBoost model outshines the rest with the highest accuracy of 94.47% while also outperforming the second-best, fast mRMR-SVM, with a score of 0.05 in accuracy. This model has a 1.04% improvement in accuracy compared to the fast mRMR-XGBoost method. Furthermore, the AdaBoost model also boasts a low FNR of 1.36% while increasing specificity to 82.69%, which solidifies this as the best methodology for this dataset.
- Fast mRMR-WSVM and fast mRMR-AdaBoost models show the highest accuracy with 94.95% in the GSE10245 dataset which beats the third model in rank fast mRMR-RF by 0.51%. Furthermore, the highest F1 Score of 96.58% with a MCC of 87.46 indicates more reliability in classification is established by fast mRMR-AdaBoost. The lowest-ranked model of all, fast mRMR-SVM, when compared to the AdaBoost model, does show a notable difference in accuracy of 4.04%, as shown. In addition, the FNR is at 0.70% making the AdaBoost method the best choice for this dataset.
- Table 2 shows the performance analysis of the model without using the BPSOA technique as the feature selection algorithm.
- Figure 3 shows the ROC analysis of the model without using BPSOA for different datasets.

### 4.2. Phase-II Evaluation

**Figure 4:**
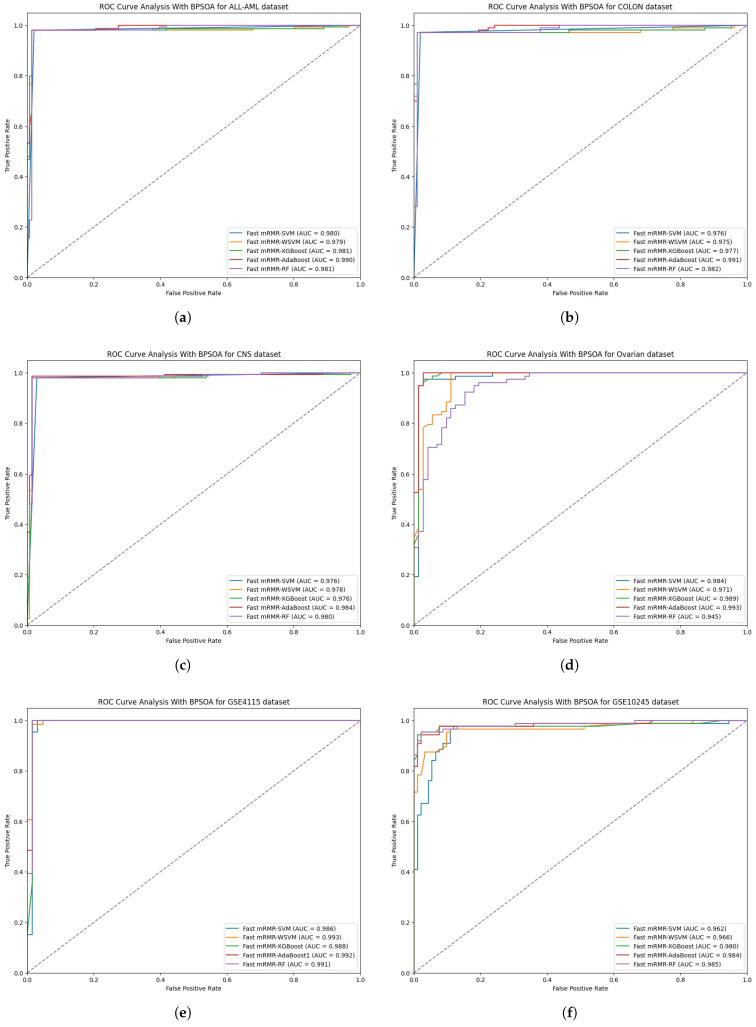
ROC analysis for (**a**) ALL-AML (**b**) colon (**c**) CNS (**d**) ovarian (**e**) GSE4115 (**f**) GSE10245 cancer datasets using Fast mRMR and BPSOA as the feature selection algorithm.

- For the ALL-MLL dataset, the leading algorithm is still the fast mRMR-BPOSA-XGBoost with an accuracy of 99.49%, F1-Score of 99.68%, and MCC score of 98.43%. This result is significantly better than the previously discussed fast mRMR-RF, with an accuracy of 93.50%, and means a 6.39% improvement.
- Similarly for the colon cancer dataset, the top performer is the fast mRMR-BPOSA-RF approach with 98.99% accuracy, F1-Score of 99.36%, and MCC of 96.92%. The previous methodology known as fast mRMR-AdaBoost was also outperformed (its accuracy was 93.94%, so the RF model shows a remarkable improvement of 5.05%). The RF model has an extremely low MCR of 1.01% and an even lower FNR of 0.64%, showing its great reliability and effectiveness in true positive detection.
- The methodology with the best results on the central nervous system dataset is the fast mRMR-BPOSA-AdaBoost, with an accuracy of 99.49%, F1-Score of 99.70%, and MCC of 98.18%. There is a noticeable improvement from the previously mentioned fast mRMR-RF (accuracy of 93.43%), with a 6.06% increase, so again there is some advantage.
- Fast mRMR-BPOSA-WSVM has an accuracy of 98.99%, F1-Score of 99.40%, and MCC of 96.18%, making it the best for the ovarian cancer dataset. Its performance is better than fast mRMR-AdaBoost, the previous best performer, which had an accuracy of 92.93%. The accuracy improvement is 6.06%, a significant increase. Furthermore, WSVM has a low FNR of 0.60%, which allows for highly accurate positive predictions.
- For the GSE4115 dataset, the best performing model is fast mRMR-BPOSA-RF with an accuracy of 99.50%, F1-Score of 99.72% and MCC of 97.32%. When comparing it to the previously mentioned model fast mRMR-AdaBoost with an accuracy of 94.47%, RF has an improvement of 5.03%. With a 0.56% FNR and a 100% perfect specificity, it is the most reliable method.
- The best model previously, fast mRMR-AdaBoost, had an accuracy of 94.95%. However, for the GSE10245 dataset, fast mRMR-BPOSA-RF has an accuracy of 97.98%, F1-Score of 98.84%, and MCC of 90.94%, which is a 3.03% increase. With an MCR of only 2.02% and a low FNR of 1.72%, for this dataset, RF proves to be the most effective methodology.
- Table 3 shows the performance analysis of the proposed model using the fast mRMR and BPSOA techniques as the feature selection algorithm.
- Figure 4 shows the ROC analysis of the proposed model using the fast mRMR and BPSOA techniques for selecting features for different datasets.

### 4.3. Critical Analysis

- The newly designed model has increased accuracy to 99.38%, which is better than any previously reported accuracy. The figure is also so much better than [5], whose accuracy was 97.23%, which is actually 2.15% better. It has also become better than what [7,8] achieved. For [7], accuracy is 98.61%, and for [8], it was 94.17%, with improvements of 0.78% and 5.21%, respectively. It can also conclude that of achieving improved accuracy of 7.98%, 2.28%, and 4.50% to the research [21,22,23], respectively. This indicates that all these improvements show the efficiency of the fast mRMR-BPSOA model while utilizing the advanced feature selection mechanism to classify the ALL-AML dataset.
- The proposed model has increased accuracy to 98.99%, which clearly shows improvement over multiple existing methods. Compared to 93.33% achieved in [5], the increase is indeed remarkable, standing at 5.66%, while compared to 93.12% garnered in [6], the number stands at 5.87%. In the same way, concerning [7] with an accuracy of 85.48% with an increment of 13.51%, while from [8] with an accuracy of 95.83%, there has been a 3.16% increase. So has it been from [11] 7.06% increment, while in the case of [20], the proposed model stands at 0.73% increment. It can also conclude that of achieving improved accuracy of 25.26%, and 3.76% to the research [21,23], respectively. These results best justify the claim of the proposed techniques concerning the different models used on the colon cancer dataset.
- The proposed model for the CNS dataset achieves an accuracy of 99.79%, again outperforming the previously best accuracy of 85.48% achieved by [7]. This phenomenal 16.46% outperformance shows that the model can perform well on complex datasets with high-dimensional features. It is also achieved improved accuracy of 20.02% to the research [21] with 83.14% of accuracy.
- The proposed model reveals that the system outperforms existing benchmarks with a 98.99% accuracy for ovarian cancer. Again, the proposed model reveals a decrement of 1.01% compared to [6,7] that scored 100%. Here, the slight increment of 0.11% reveals the model outperforms 98.88% from [11]. Also, it outperforms 96.98 from [20] with a 2.01% increment. It is also achieved improved accuracy of 5.17% to the research [21] with 94.10% of accuracy. The developed model seems to perform reasonably well but does not outperform all benchmark strategies for the ovarian cancer dataset.
- For the GSE4115 dataset the model’s accuracy on the GSE4115 dataset is 99.50%, which is also the improvements in accuracy of 8.76% to [21] with 91.49% of accuracy. The accuracy on the GSE10245 dataset is 97.98%, which is also an improved accuracy of 11.04% and 8.75% to the research [21,23], respectively. In any case, these results illustrate the strength of the proposed model in working across different datasets.
- Table 4 shows the comparative study of the proposed model with existing models.

## 5. Conclusions

This research introduces an advanced methodology for cancer diagnosis that hybridizes machine learning techniques with feature extraction processes, integrating fast mRMR with Portia spider optimization. The incorporation of mRMR with Portia spider optimization BPOSA extracted the most relevant and least associated redundant features, which increased classification performance while minimizing the costs incurred by the computations. This process is performed on the classifying algorithms SVM, weighted SVM, XGBoost, AdaBoost, and Random Forest (RF). The method above was applied to six diverse cancer datasets: ALL-MLL, colon, CNS, ovarian, GSE4115, and GSE10245. In terms of classification, the fast mRMR-BPOSA-AdaBoost was overwhelmingly the best performer achieving an astounding 99.79% accuracy. These remarkable achievements suggest that this AdaBoost algorithm is the best approach for accurate cancer classification given mRMR feature selection and is the best for high-dimensional genomic datasets. These results emphasize the potential of the proposed hybrid machine learning framework to tackle the challenges such as high-dimensionality, feature redundancy, and imbalance that are posed in cancer prognosis and final decision making diagnosis support systems (DSSs) for cancer detection. With such astounding accuracy, it is apparent that this model overcomes the primary challenge that detection and diagnosis models face, which is the robustness of the model allowing it to aid and support treatment actions and planning in a timely manner. Future work might delve into cross-omics datasets and the use of this model in actual clinical cases with an effort toward personalized medicine and to develop a parallel computational model using fast mRMR and BPOSA.

## Figures and Tables

**Table 1:** Summary of biomedical datasets.

Dataset	Instances/Attributes	Classes Distribution	Imbalance Ratio
A1	70/7129	AML/ALL: 25/47	1.88
A2	62/2000	Tumor/Normal: 40/22	1.82
A3	60/7129	Class0/Class1: 39/21	1.85
A4	235/15,154	Cancer/Normal: 162/91	1.78
A5	192/22,216	Cancer/Normal: 97/95	1.02
A6	58/54,676	AC/SCC: 40/18	2.22

**Table 2:** Performance metrics for different datasets using fast mRMR with various classifiers.

Dataset	Model	$A_{\mathrm{cc}}$ (%)	MCR (%)	$P_{\mathrm{re}}$ (%)	$R_{\mathrm{cl}}$ (%)	$F_{1\mathrm{Sc}}$ (%)	$S_{\mathrm{pe}}$ (%)	${\mathrm{Fa}}_{\mathrm{Neg}}$ (%)	${\mathrm{Fa}}_{\mathrm{Po}}$ (%)	MCC (%)
ALL-AMLL	Fast mRMR-SVM	88.89	11.11	85.27	97.35	90.91	77.65	2.65	22.35	77.90
	Fast mRMR-WSVM	90.91	9.09	86.29	99.07	92.24	81.11	0.93	18.89	82.53
	Fast mRMR-XGBoost	91.41	8.59	91.74	94.07	92.89	87.50	5.93	12.50	82.10
	Fast mRMR-AdaBoost	92.89	7.11	91.27	97.46	94.26	86.08	2.54	13.92	85.27
	Fast mRMR-RF	93.50	6.50	91.47	98.33	94.78	86.25	1.67	13.75	86.60
COLON	Fast mRMR-SVM	88.38	11.62	86.61	94.83	90.53	79.27	5.17	20.73	76.10
	Fast mRMR-WSVM	93.43	6.57	90.55	99.14	94.65	85.37	0.86	14.63	86.79
	Fast mRMR-XGBoost	92.93	7.07	93.39	94.96	94.17	89.87	5.04	10.13	85.21
	Fast mRMR-AdaBoost	93.94	6.06	93.02	97.56	95.24	88.00	2.44	12.00	87.10
	Fast mRMR-RF	91.92	8.08	91.13	95.76	93.39	86.25	4.24	13.75	83.18
CNS	Fast mRMR-SVM	87.37	12.63	86.40	93.10	89.63	79.27	6.90	20.73	73.89
	Fast mRMR-WSVM	88.38	11.62	87.80	93.10	90.38	81.71	6.90	18.29	75.97
	Fast mRMR-XGBoost	91.41	8.59	88.52	97.30	92.70	83.91	2.70	16.09	82.87
	Fast mRMR-AdaBoost	92.42	7.58	91.27	96.64	93.88	86.08	3.36	13.92	84.20
	Fast mRMR-RF	93.43	6.57	92.74	96.64	94.65	88.61	3.36	11.39	86.29
Ovarian	Fast mRMR-SVM	89.90	10.10	90.07	95.49	92.70	78.46	4.51	21.54	76.70
	Fast mRMR-WSVM	91.92	8.08	92.48	95.35	93.89	85.51	4.65	14.49	82.04
	Fast mRMR-XGBoost	92.42	7.58	89.60	98.25	93.72	84.52	1.75	15.48	84.79
	Fast mRMR-AdaBoost	92.93	7.07	94.20	95.59	94.89	87.10	4.41	12.90	83.44
	Fast mRMR-RF	90.82	9.18	90.00	94.74	92.31	85.37	5.26	14.63	81.10
GSE4115	Fast mRMR-SVM	94.42	5.58	93.06	99.26	96.06	83.87	0.74	16.13	87.06
	Fast mRMR-WSVM	93.94	6.06	92.03	99.22	95.49	84.29	0.78	15.71	86.87
	Fast mRMR-XGBoost	93.43	6.57	93.88	97.18	95.50	83.93	2.82	16.07	83.54
	Fast mRMR-AdaBoost	94.47	5.53	94.16	98.64	96.35	82.69	1.36	17.31	85.42
	Fast mRMR-RF	93.43	6.57	91.95	99.28	95.47	80.00	0.72	20.00	84.42
GSE10245	Fast mRMR-SVM	90.91	9.09	92.95	95.39	94.16	76.09	4.61	23.91	73.84
	Fast mRMR-WSVM	94.95	5.05	96.88	96.88	96.88	86.84	3.13	13.16	83.72
	Fast mRMR-XGBoost	93.94	6.06	95.63	96.84	96.23	82.50	3.16	17.50	80.89
	Fast mRMR-AdaBoost	94.95	5.05	94.00	99.30	96.58	83.93	0.70	16.07	87.46

**Table 3:** Performance metrics for different datasets using fast mRMR-BPSOA with various classifiers.

Dataset	Model	$A_{\mathrm{cc}}$ (%)	MCR (%)	$P_{\mathrm{re}}$ (%)	$R_{\mathrm{cl}}$ (%)	$F_{1\mathrm{Sc}}$ (%)	$S_{\mathrm{pe}}$ (%)	${\mathrm{Fa}}_{\mathrm{Neg}}$ (%)	${\mathrm{Fa}}_{\mathrm{Po}}$ (%)	MCC (%)
ALL-AML	Fast mRMR-BPSOA-SVM	95.48	4.52	95.48	98.67	97.05	85.71	1.33	14.29	87.60
	Fast mRMR-BPSOA-WSVM	97.47	2.53	97.44	99.35	98.38	91.11	0.65	8.89	92.73
	Fast mRMR-BPSOA-XGBoost	99.38	0.62	99.37	100.00	99.68	97.50	0.00	2.50	98.43
	Fast mRMR-BPSOA-AdaBoost	98.48	1.52	98.74	99.37	99.05	95.00	0.63	5.00	95.27
	Fast mRMR-BPSOA-RF	98.99	1.01	99.38	99.38	99.38	97.30	0.62	2.70	96.68
COLON	Fast mRMR-BPSOA-SVM	97.98	2.02	99.39	98.20	98.80	96.77	1.80	3.23	92.61
	Fast mRMR-BPSOA-WSVM	96.97	3.03	98.11	98.11	98.11	92.31	1.89	7.69	90.42
	Fast mRMR-BPSOA-XGBoost	97.98	2.02	99.38	98.17	98.77	97.06	1.83	2.94	93.12
	Fast mRMR-BPSOA-AdaBoost	98.48	1.52	99.39	98.79	99.09	96.97	1.21	3.03	94.63
	Fast mRMR-BPSOA-RF	98.99	1.01	99.36	99.36	99.36	97.56	0.64	2.44	96.92
CNS	Fast mRMR-BPSOA-SVM	95.96	4.04	97.62	97.62	97.62	86.67	2.38	13.33	84.29
	Fast mRMR-BPSOA-WSVM	97.98	2.02	100.00	97.63	98.80	100.00	2.37	0.00	92.63
	Fast mRMR-BPSOA-XGBoost	97.34	2.66	99.35	97.45	98.39	96.77	2.55	3.23	90.85
	Fast mRMR-BPSOA-AdaBoost	99.79	0.21	99.40	100.00	99.70	96.97	0.00	3.03	98.18
	Fast mRMR-BPSOA-RF	97.98	2.02	99.39	98.19	98.79	96.88	1.81	3.13	92.79
Ovarian	Fast mRMR-BPSOA-SVM	97.47	2.53	98.74	98.13	98.43	94.74	1.88	5.26	91.95
	Fast mRMR-BPSOA-WSVM	98.99	1.01	99.40	99.40	99.40	96.77	0.60	3.23	96.18
	Fast mRMR-BPSOA-XGBoost	97.98	2.02	98.85	98.85	98.85	91.67	1.15	8.33	90.52
	Fast mRMR-BPSOA-AdaBoost	98.48	1.52	99.43	98.87	99.15	95.24	1.13	4.76	92.21
	Fast mRMR-BPSOA-RF	96.95	3.05	98.16	98.16	98.16	91.18	1.84	8.82	89.34
GSE4115	Fast mRMR-BPSOA-SVM	97.47	2.53	98.20	98.80	98.50	90.63	1.20	9.38	90.58
	Fast mRMR-BPSOA-WSVM	99.49	0.51	100.00	99.39	99.70	100.00	0.61	0.00	98.22
	Fast mRMR-BPSOA-XGBoost	98.99	1.01	99.39	99.39	99.39	96.97	0.61	3.03	96.36
	Fast mRMR-BPSOA-AdaBoost	97.47	2.53	99.37	97.53	98.44	97.22	2.47	2.78	91.89
	Fast mRMR-BPSOA-RF	99.50	0.50	100.00	99.44	99.72	100.00	0.56	0.00	97.32
GSE10245	Fast mRMR-BPSOA-SVM	94.44	5.56	97.59	95.86	96.72	86.21	4.14	13.79	78.83
	Fast mRMR-BPSOA-WSVM	96.97	3.03	98.29	98.29	98.29	86.96	1.71	13.04	85.24
	Fast mRMR-BPSOA-XGBoost	96.97	3.03	98.16	98.16	98.16	91.43	1.84	8.57	89.59
	Fast mRMR-BPSOA-AdaBoost	96.46	3.54	99.40	96.51	97.94	96.15	3.49	3.85	86.13
	Fast mRMR-BPSOA-RF	97.98	2.02	99.42	98.28	98.84	95.83	1.72	4.17	90.94

**Table 4:** Performance comparison (proposed vs. existing models) (%).

Methodology	ALL-AML	Colon	CNS	Ovarian	GSE4115	GSE10245
[5]	97.23	93.33	–	–	–	–
[6]	–	93.12	–	100	–	–
[7]	98.61	85.48	83.33	100	–	–
[8]	94.17	95.83	–	–	–	–
[11]	–	91.93	–	98.88	–	–
[20]	–	98.27	–	96.98	–	–
[21]	92.11	79.03	83.14	94.10	91.49	88.24
[22] with RNN	97.11	–	–	–	–	–
[22] with LSTM	97.17	–	–	–	–	–
[23] with CNN	94.50	93.60	–	–	–	87.70
[23] with LSTM	94.10	94.30	–	–	–	88.20
[23] with DCNN	95.10	95.40	–	–	–	90.10
**[Proposed]**	**99.38**	**98.99**	**99.79**	**98.99**	**99.5**	**97.98**

## Data Availability

The dataset used in the manuscript is available at http://csse.szu.edu.cn/staff/zhuzx/Datasets.html and https://www.ncbi.nlm.nih.gov/geo accessed on 14 December 2024.
